# Diagnosis and potential invasion risk of *Thrips parvispinus* under current and future climate change scenarios

**DOI:** 10.7717/peerj.13868

**Published:** 2022-08-25

**Authors:** Timmanna Hulagappa, Gundappa Baradevanal, Shwetha Surpur, Devaramane Raghavendra, Sagar Doddachowdappa, Pathour R. Shashank, Kumaranag Kereyagalahalli Mallaiah, Jamuna Bedar

**Affiliations:** 1 Division of Entomology, Indian Agricultural Research Institute, New Delhi, Delhi, India; 2 Crop Protection, ICAR-Central Institute for Subtropical Horticulture, Lucknow, Uttar Pradesh, India; 3 Directorate of Plant Protection Quarantine and Storage, Central IPM Centre, Jeedimetla, Hyderabad, Telangana, India; 4 Entomology, ICAR-National Research Centre for Integrated Pest Management, New Delhi, Delhi, India; 5 PRFQAL Lab Agricultural Entomology, University of Agricultural Sciences Raichur, Raichur, Karnataka, India

**Keywords:** Chilli, Climate change, Distribution, Diversity, Ecological niche modelling, Invasive species, Potential risk, Thrips parvispinus, Prediction, Yield loss

## Abstract

**Background and Objective:**

Invasive thrips, *Thrips parvispinus* Karny recently reported in India, causing a widespread severe infestation in more than 0.4 million ha of chilli (*Capsicum annum* L.) growing areas. This species is native to Thailand and most prevalent in other South East Asian countries. Large scale cultivation of the major host plants (chilli and papaya), and favourable climatic conditions in India and other countries similar to native range of *Thrips parvispinus* expected to favour its further spread and establishment to new areas.

**Materials and Methods:**

The present study was undertaken to confirm invasive thrips species identity through both morphological and molecular approaches and predict its potential invasion using the maximum entropy (MaxEnt) algorithm.

**Results:**

The model predicted species range in respect of discrimination of suitable and unsuitable areas for its occurrence both in current and future climatic scenarios. The model provided a good fit for species distribution with a high value of area under the curve (0.957). The jackknife test indicated annual mean temperature and precipitation were found to be the most important bioclimatic variable in determining the distribution of *T. parvispinus*. High suitability areas were predicted in the countries wherever its occurrence was reported with high discrimination ability of suitable and unsuitable areas. Key distinguishing morphological characters of *T. parvispinus* were illustrated through high-resolution scanning electron microscopic images.

**Conclusion:**

The identity of the thrips causing wide spread damage in chilli confirmed through morphological and molecular approaches. Key identifying characters were described through high resolution scanning electron microscopic images for accurate identification of the species. MaxEnt model identified high suitability regions for the potential establishment of *T. parvispinus* in India and other parts of the world. This study facilitates forecasting of further spread and also suggests imposing strict domestic quarantine measures to curtail its establishment in the new areas.

## Introduction

Biological invasions cause a greater negative impact on the native ecosystem and biodiversity. The factors contributing to successful invasion remain elusive which leads to severe ecological and economic damage. Further, extreme weather events triggered by the climate change may enhance invasion processes, from its initial introduction to the establishment and spread (Diez et al., 2012).

In the era of climate change, forecasting of occurrence and spread of potential invasive pests is of considerable interest for better handling and management of invasive species (Kumar et al., 2015; Fand et al., 2020). Ecological niche modelling (ENM) is a rapidly evolving field for estimating the distribution of species under different climate change scenarios (Peterson, 2011). Several methods have been used for predicting spatial distribution based on species presence data with environmental variables (Guisan & Zimmermann, 2000; Elith et al., 2006). One of the most common methods nowadays in use is the maximum entropy (MaxEnt) model (Halvorsen et al., 2015), which estimates a probability distribution of intended species (Phillips, Anderson & Schapire, 2006; Phillips & Dudík, 2008; Elith et al., 2011).

Integration of species distribution models and ENM is a more appropriate approach to predict the potential distribution and spread of various pests based on occurrence records and corresponding climatic and other environmental variables (Bentlage et al., 2013; Kumar, Neven & Yee, 2014). ENM has been used widely in biological invasion, conservation biology, biological responses to climate change, and various aspects of ecological and evolutionary biological research (Peterson, Bauer & Mills, 2004). Recently, mapping of the potential distribution of agriculturally important insect pests using maximum entropy modelling is extensively being used for suitability assessment and risk zoning of invasive species (Wang et al., 2020; Baloch et al., 2020; Baradevanal et al., 2021).

Thrips (Thysanoptera) are presumed to have evolved from fungus-feeding detritus-living ancestors (Mound, 2002), and subsequently adopted three major food sources *viz*., fungal hyphae and spores, green leaves, and flowers. A few species are predators, and very few feed on mosses (Mound & Marullo, 1996). So far, 739 thrips species recorded from India (Tyagi & Kumar, 2016), of which five species *viz*. *Scirtothrips dorsalis* Hood, *Thrips palmi* Karny *Thrips tabaci* Lindman, *Frankliniella schultzie* Trybom, *Ceratothripoides claretris* reported as vectors tospoviruses, causing 10 to 60 percent yield loss in various solanaceous, cucurbitaceous, and leguminaceous crops (Singh & Krishnareddy, 1996; Meena et al., 2005; Gopal et al., 2010; Mandal et al., 2012).

The recently invaded thrips, *Thrips parvispinus* (Karny) originated from Thailand (Mound & Collins, 2000) and is considered a destructive polyphagous pest causing significant damage to pepper (*Capsicum annum*) and other solanaceous crops in Indonesia (Vos, 1994; Murai, 2002; Sartiami & Mound, 2013; Johari, 2015). The increased movement of plant materials through international trade made *T. parvispinus* spread rapidly to other Southeast Asian countries including Oceania, North America, Europe, Africa, and now India (Waterhouse, 1993; Zhang, Xie & Li, 2011; Table S1). The last two decades witnessed a drastic expansion in the geographic distribution and host range. *Thrips parvispinus* (Karny) causes significant damage to pepper (*Capsicum annum*) resulting in yield loss of up to 23 per cent reported under field conditions in Indonesia (Johari & Natalia, 2018; NPPO, 2019), causing severe economic damage in papaya at Hawaii and other North American states (Sugano et al., 2013). In Malaysia, *T. parvispinus* feeding injury attracted saprophytic fungus (*Cladosporium oxysporum*) causing ‘bunchy top’ malformation in papaya (Lim, 1989). In Spain, serious damage was observed in crops (*Dipladenia, Gardenia, Citrus*) cultivated in greenhouses.

In India, *Thrips parvispinus* (Karny) infestation was first time reported on papaya (*Carica papaya* L) (Tyagi et al., 2015) and subsequently on various host plants *viz*., *Brugmansia* sp., *Tagetes* sp., *Citrulluslanatus* (Thunb.) *Momordica charantia* L. *Chrysanthemum* sp., *Gossypium* sp., *Mangifera indica* L. *Tamarindus indica* L. and *Dahlia rosea*, *Capsicum annum* (Rachana et al., 2021; Roselin, Sharma & Rachana, 2021; Nagaraju et al., 2021). As per the joint survey report by the Directorate of Plant Protection Quarantine and Storage and State Agriculture Universities and Agriculture Departments of Telangana and Andhra Pradesh, more than 0.4 m ha of chilli crop infested with this invasive menace and 10 to 30 per cent yield loss was observed. A higher level of infestation (10 to 20 thrips/flower) was noticed in the Warangal, Khammam, and Guntur districts of Andhra Pradesh (Janyala, 2021; Directorate of Plant Protection Quarantine and Storage of India, 2021).

The accurate identification of the pest species is the basic step for pest risk assessment programs. Thrips are minute insects (0.2 to 5 mm), the reliable identification of a species requires an expert taxonomist. The inappropriate identification results confused species identification of potential pests with native non-pest species like *Thrips palmi* as *Thrips flavus* in India (Singh & Krishnareddy, 1996). *Thrips parvispinus* as *Thrips (Isoneurothrips) taiwanus* Takahashi in Netherland (Mound & Collins, 2000). The ongoing changes to the climate in the Indian peninsula might facilitate the chance for the establishment and geographical expansion of exotic insect pests, especially those from tropical climates. India is one of the leading producers of chilli and papaya and the second-largest producer of vegetables in the world, further spread, and the outbreak of this invasive pest may pose a serious threat to the Indian and global economies. In this regard, the correct identification of invasive *T. parvispinus* was ascertained through the morphological and molecular approach and further analyzed potential distribution in current and future climatic conditions.

## Materials and Methods

The roving survey was conducted in *T. parvispinus* invaded major chilli-growing districts of south Indian states (Andhra Pradesh, Telangana, and Karnataka) during November and December 2021 (Table 1). Three villages were selected from each district and observations were made on the number of thrips per plant and their percent damage. The terminal leaves and flowers of infested plants were tapped on A4 size white paper and fallen thrips were collected in vials containing 70% ethanol by using a fine camel hair brush and used for morphological taxonomy (slide mounts and scanning electron microscopic photography) and further confirmed with the molecular approach.

**Table 1:** Thrips parvispinus infestation in South Indian states surveyed for *T. parvispinus* Karny.

State	Name of the district	Infestation level (Mean no of thrips/flower)
Andhra Pradesh	Guntur	20.25
	Prakasham	14.13
	Krishna	11.50
	Rajahmundry	12.00
Telangana	Khammam	19.00
	Kothagudem	7.13
	Mehabubabad	19.00
	Warangal	18.88
Karnataka	Bellary	10.13
	Koppal	7.38
	Chitradurga	5.50
	Gadag	5.00
	Raichur	6.75

### Morphological diagnosis of *T. parvispinus*

Thrips samples collected in 70% ethanol were dehydrated in a series of ethanol grades and 50% and 100% of drying mixture (Hexamethyldisilizane:ethanol) (Kumar et al., 2011). After dehydration steps, the specimens were mounted on aluminum stubs coated with 24 nm palladium. Subsequently, examined under Scanning Electron Microscope (SEM) (VEGA3 TESCAN SEM at 10 kV 10 pa Czech Republic) at a magnification of 2 µm to 100 µm. The microscopic slide mounts of thrips were prepared by following standard maceration and dehydration protocol (Ananthakrishnan & Sen, 1980). The morphological diagnosis was carried out under an optical microscope (DM2500 LED) using standard morphological keys (Hoddle, Mound & Paris, 2012). The voucher specimens were submitted to National Pusa Collection, ICAR-Indian Agricultural Research Institute, New Delhi, India.

### Molecular diagnosis of *T. parvispinus*

For molecular identification, genomic DNA was isolated from field-collected thrips samples from surveyed locations using DNeasy Blood and Tissue Kit (Qiagen GmbH, Hilden, Germany) by following the manufacturer’s protocol and visualized on 0.8% agarose gel in UV Gel Documentation system (Alpha Imager HP; Protein Simple, San Jose, CA, USA). The PCR product amplification, purification, sequence alignment, and analysis were performed as per the methodology described by Shashank, Thomas & Ramamurthy (2015). The amplified PCR products were eluted using a gel elution kit and purified using a QIAquick PCR purification kit (Qiagen GmbH, Hilden, Germany) as per the manufacturer’s protocol. The purified PCR products were sequenced in both directions (ABI prism® 3730 XL DNA Analyzer; Applied Biosystems, Waltham, MA, USA) at Green Genome India Private Limited, Delhi. After obtaining the sequences in both the directions, they were aligned using the sequence alignment editor BioEdit version 7.0.5.3 (Hall, 1999), and the aligned sequences were then queried against the online ‘Nucleotide BLAST’ blastn tool of the National Center for Biotechnology Information (NCBI) to confirm the species identity, later the sequences were deposited with the NCBI database and the accession numbers were obtained.

Along with our sequence (OM640391), three other sequences (KM485666, OM085663, KF144125) were retrieved from NCBI database. *Thrips hawaiiensis* (MZ569053.1) was used as it is a member of the Thrips genus and *Frankliniella schultzie* (KC513151.1) was used as an outgroup. Phylogenetic relationship was inferred by applying a Bayesian analysis of molecular sequences using Markov chain Monte Carlo (MCMC) phylogenetic search using BEAST v1.10.4 software as per the methodology described by Suchard et al. (2018). A general time-reversible (GTR) model was selected for the COI sequences analysis and other default parameters. The tree was visualized and edited using FigTree v1.4.4 (http://tree.bio.ed.ac.uk/software/figtree/).

### Pest occurrence records

**Figure 1:**
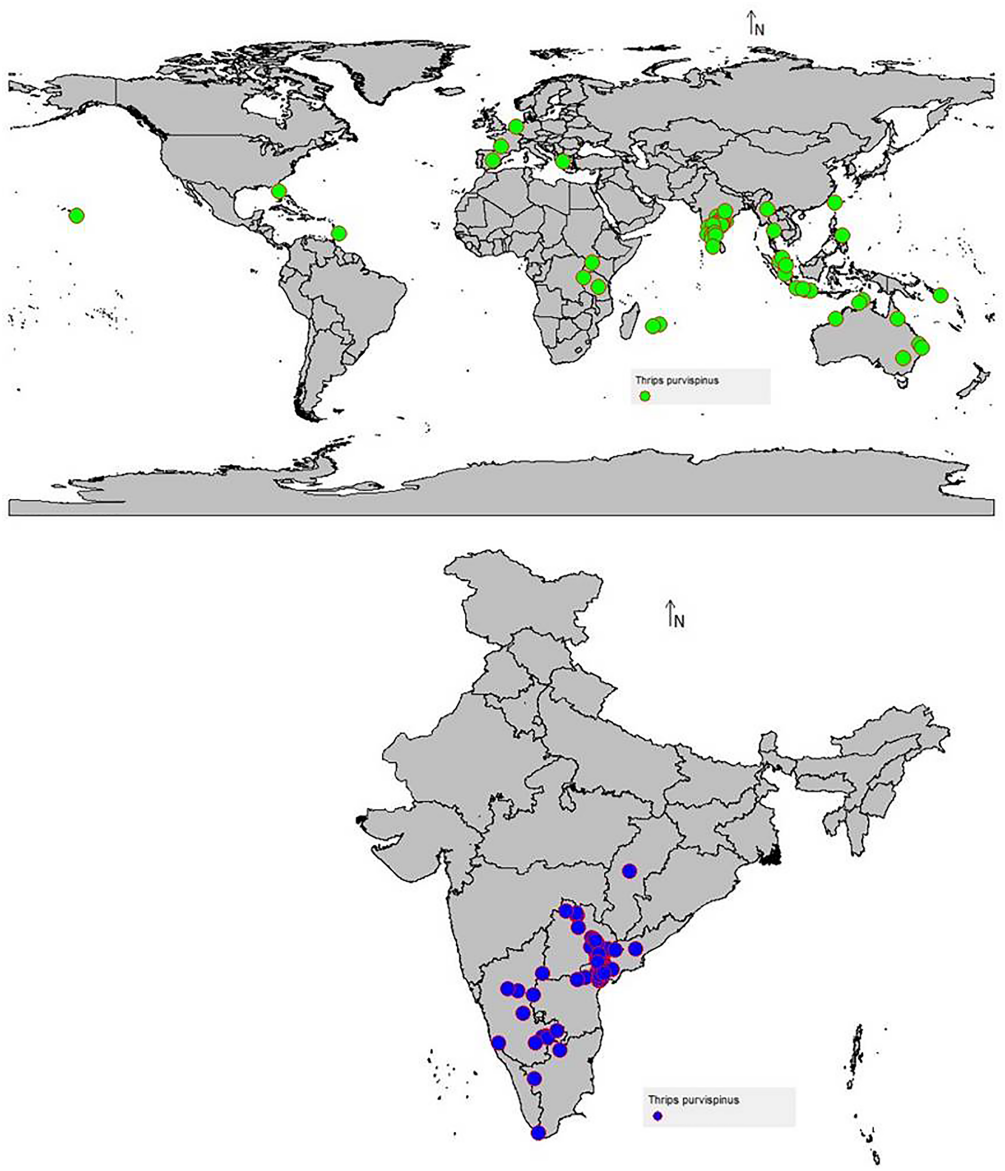
*Thrips parvispinus* occurrence records of India and the world.

The data on the current distribution of invasive *T. parvispinus* were collected during the survey in major chilli growing states (Andhra Pradesh, Karnataka, Telangana) of India and global occurrence records were obtained by data mining of published literature and databases (CABI, 2021; EPPO, 2022). A total of 88 valid occurrence records were used in the study (Fig. 1). To reduce the uncertainty and sample biasness, duplicate records and neighboring occurrence points were removed by ‘spThin’ R package as previously described by Baradevanal et al. (2021).

### Current and future climate data

Current data of 19 bioclimatic variables with a resolution at 2.5 m were retrieved from World clim data base version 1.4 (http://www.worldclim.org/). To meet the requirement of the study, the data were further processed and muticollinearity among the bioclimatic variables was verified as suggested by Baradevanal et al. (2021). Highly correlated bio-climatic variables were determined by using Pearson correlation coefficient through pair-wise comparisons. The varibles with Pearson’s coefficient (r) value of ≥0.80, were retained for model development considering its predictive power (*i.e.*, percent contribution and jackknife gain). Accordingly, the best describing nine bioclimatic variables were further processed that included, annual mean temperature, mean diurnal range, isothermality, temperature seasonality, maximum temperature of warmest month, annual precipitation), precipitation of wettest month, precipitation of driest month and precipitation of seasonality were used in the study. Statistically downscaled and bias-corrected future climate data were obtained from Climate Change Agriculture and Food Security (CCAFS) (http://www.ccafs-climate.org) at a spatial resolution of 2.5. The Global Climate Model (GCM) of the Commonwealth Scientific and Industrial Research Organization (CSIRO.MK3) represents simulations for two representative concentration pathways (RCP 2.6 and RCP 8.5) from the Fifth Assessment of the Intergovernmental Panel for Climate Change (CMIP5) was selected for representing the future climatic scenario by the year 2030, 2050, 2070 and 2080. Each scenario represents the radiative force estimated for future climate based on the predicted greenhouse gas emissions.

### Model development and evaluation

Maximum entropy (MaxEnt V. 3.4.1) was used for species distribution modelling. It is a machine learning algorithm, that estimates the probability of distribution of a species. MaxEnt was chosen because it uses species presence only and background data. The model settings were employed as convergence threshold (10–5), maximum iterations (5,000), and the maximum number of background points (10,000) to run the model. Based on background data, the jackknife test was performed to estimate the relative importance of each of the variables to the model development. The model performance was validated using the area under the curve (AUC) of the receiver operating characteristic (ROC) with 25% of the localities randomly selected to test the model. The AUC values below 0.5 are interpreted as a random prediction. Whereas values 0.5 and 0.7 indicate poor model performance, 0.7 to 0.9 indicates reasonable performance, and >0.9 indicates high model performance (Pearson et al., 2007). Predicted distribution maps were processed as per the procedure described by Baradevanal et al. (2021).

## Results

**Figure 2:**
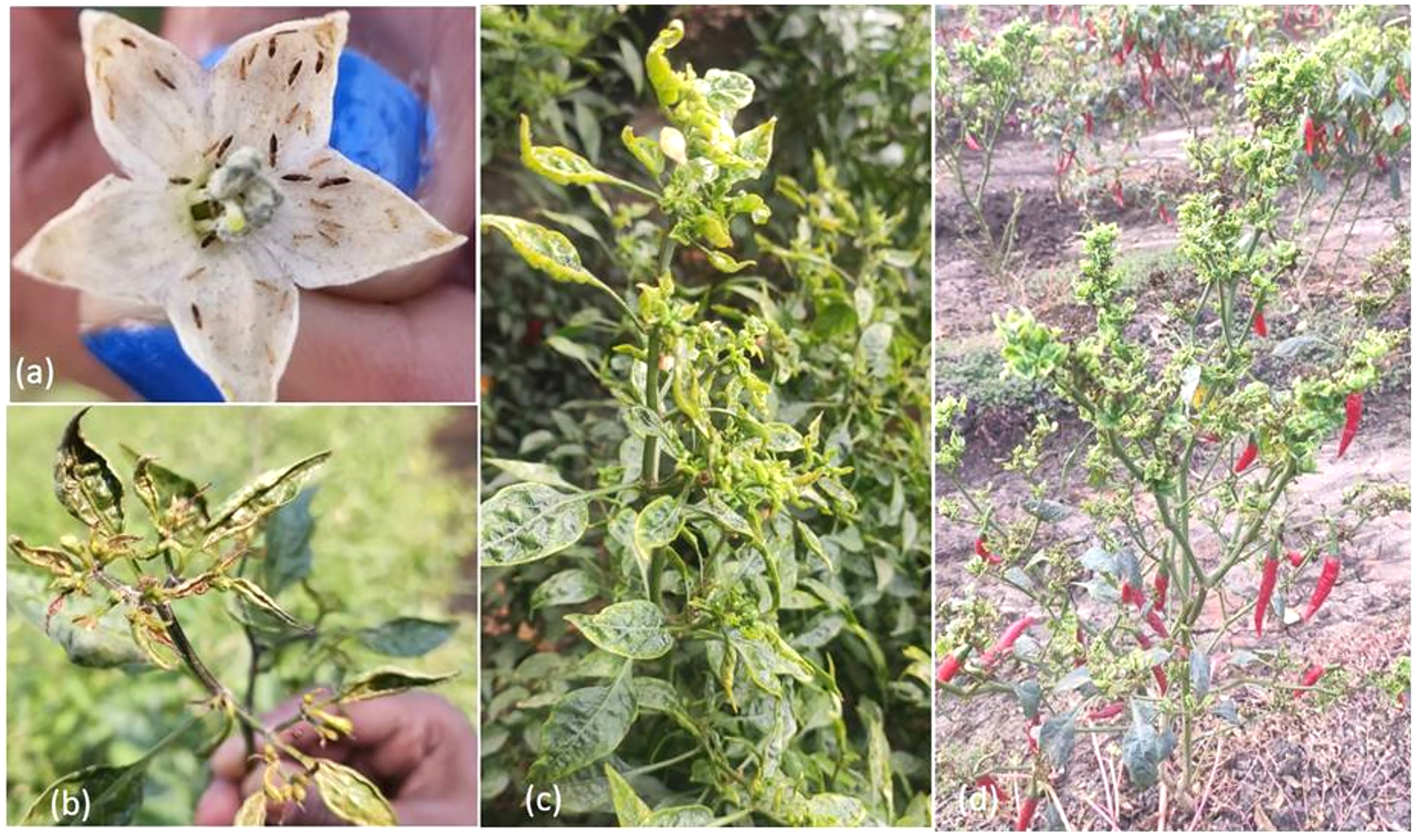
*Thrips parvispinus* damage symptoms in chilli crop. (A) Thrips feeding on flowers, (B) damaged shoot buds, (C) curling and crinkling of leaves, (D) new flesh in infested plants.

The roving survey revealed moderate to severe infestation of thrips during the flowering to fruiting stage chilli crop (90 to 120 days old). More than 60% of the chilli-growing area was affected in major chilli growing districts of Andhra Pradesh, Telangana, and Karnataka, and more than 90% area of chilli area was covered with hybrids. Thrips preferred to feed inside flowers, immature and adult thrips resided all along midribs at the lower surface of leaves. Among the districts surveyed, a higher level of infestation (10 to 20 thrips/flower) was recorded in Guntur (Andhra Pradesh), Mahabubabad, and Warangal (Telangana) districts (Table 1). Infested plants showed damage symptoms *viz*., silvery appearance to brownish discoloration on tender leaves and fruits, crinkling and upward curling of leaves with elongated petioles, buds and leaves turned brittle, and also a large number of flowers dropping and necrosis of new flesh was observed (Fig. 2).

### Morphological diagnosis of thrips

**Figure 3:**
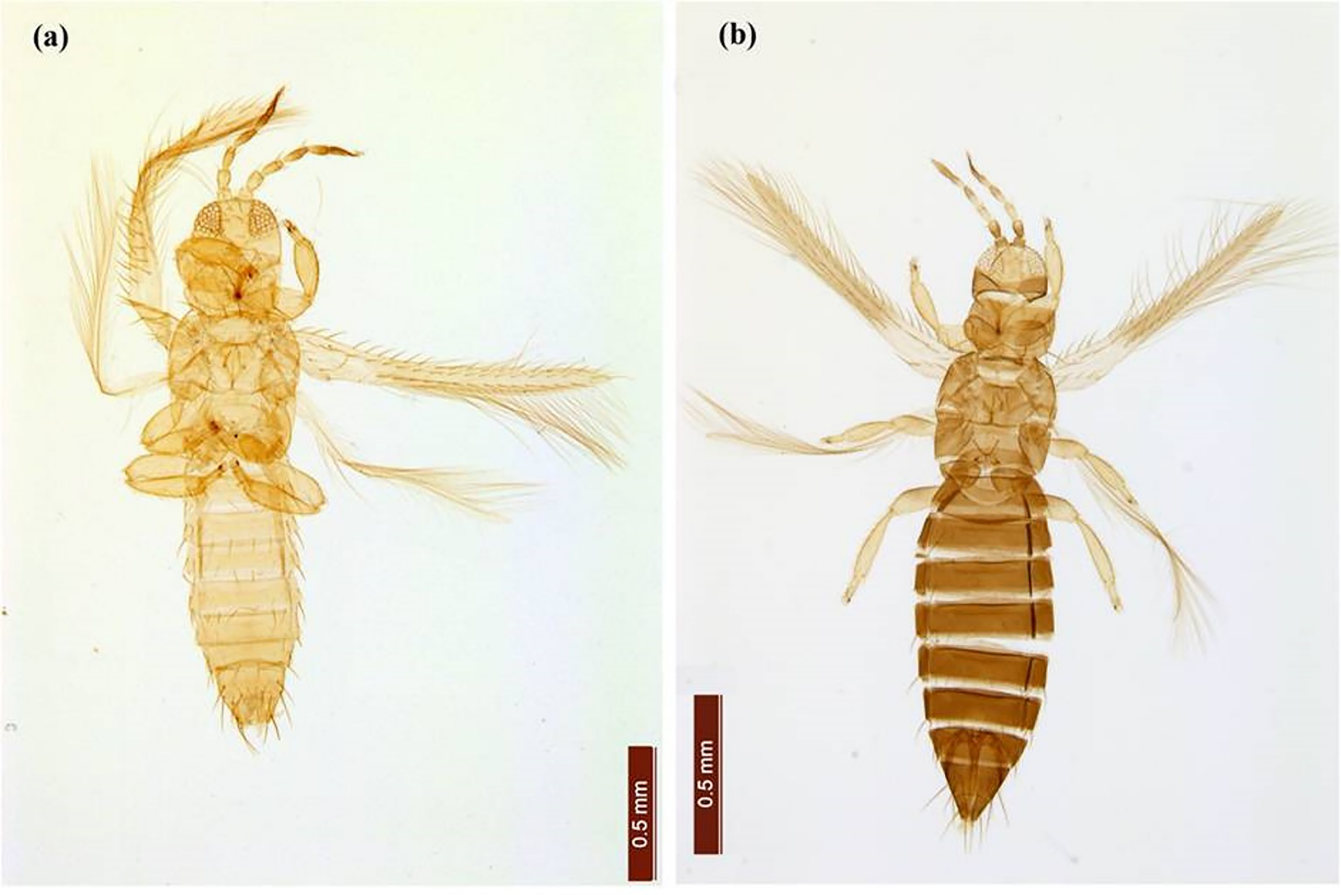
*Thrips parvispinus adults*. (A) Male, (B) female.

**Figure 4:**
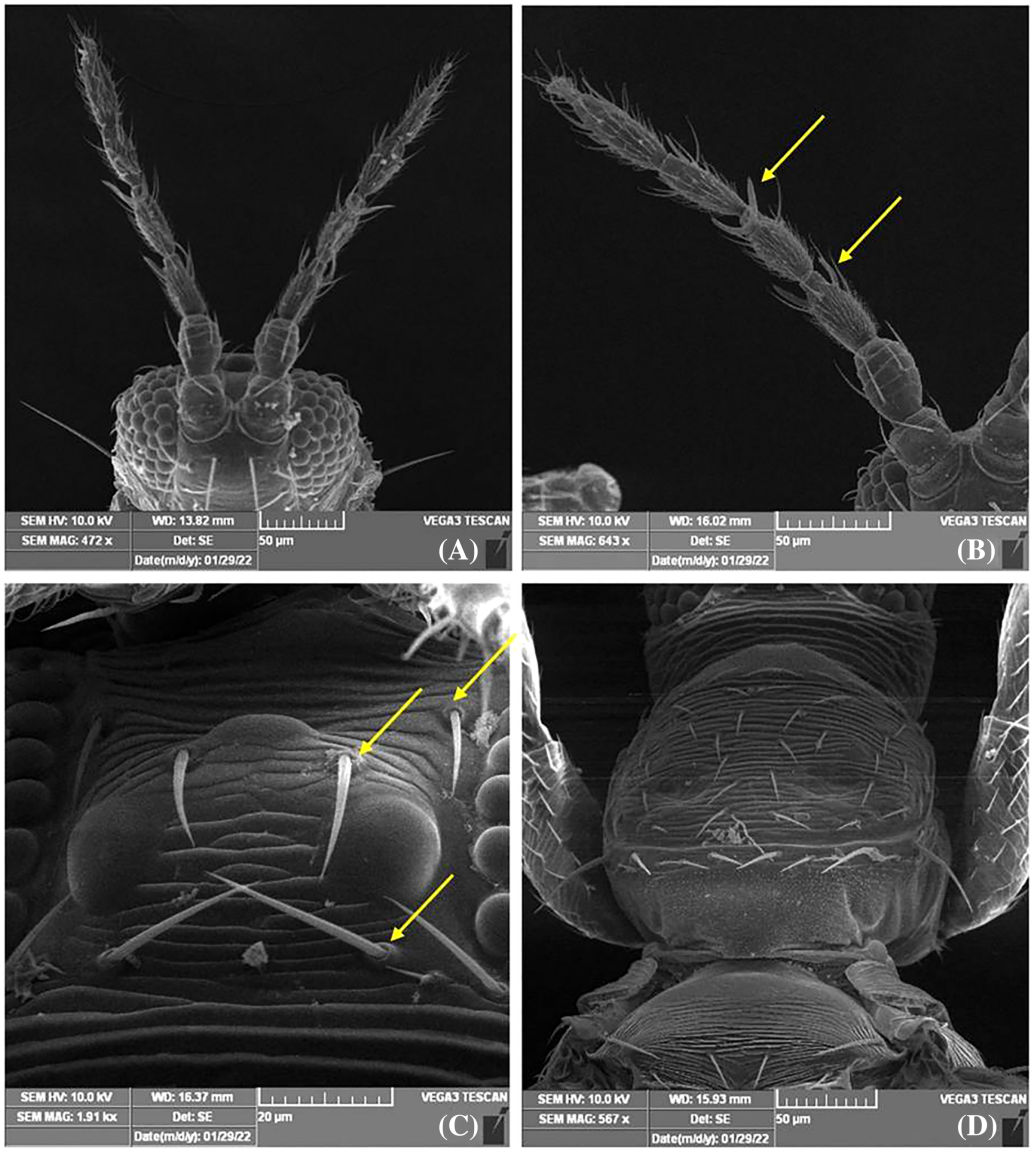
SEM images of *Thrips parvispinus*. (A) Antennae 7 segmented; (B) forked sensory organs present on III & IV segments; (C) three pairs of ocellar setae, pair III is small and arising on anterior margins of ocellar triangle; (D) pronotum with two pairs of long posteroangular setae and posterior margin with three pairs of setae.

**Figure 5:**
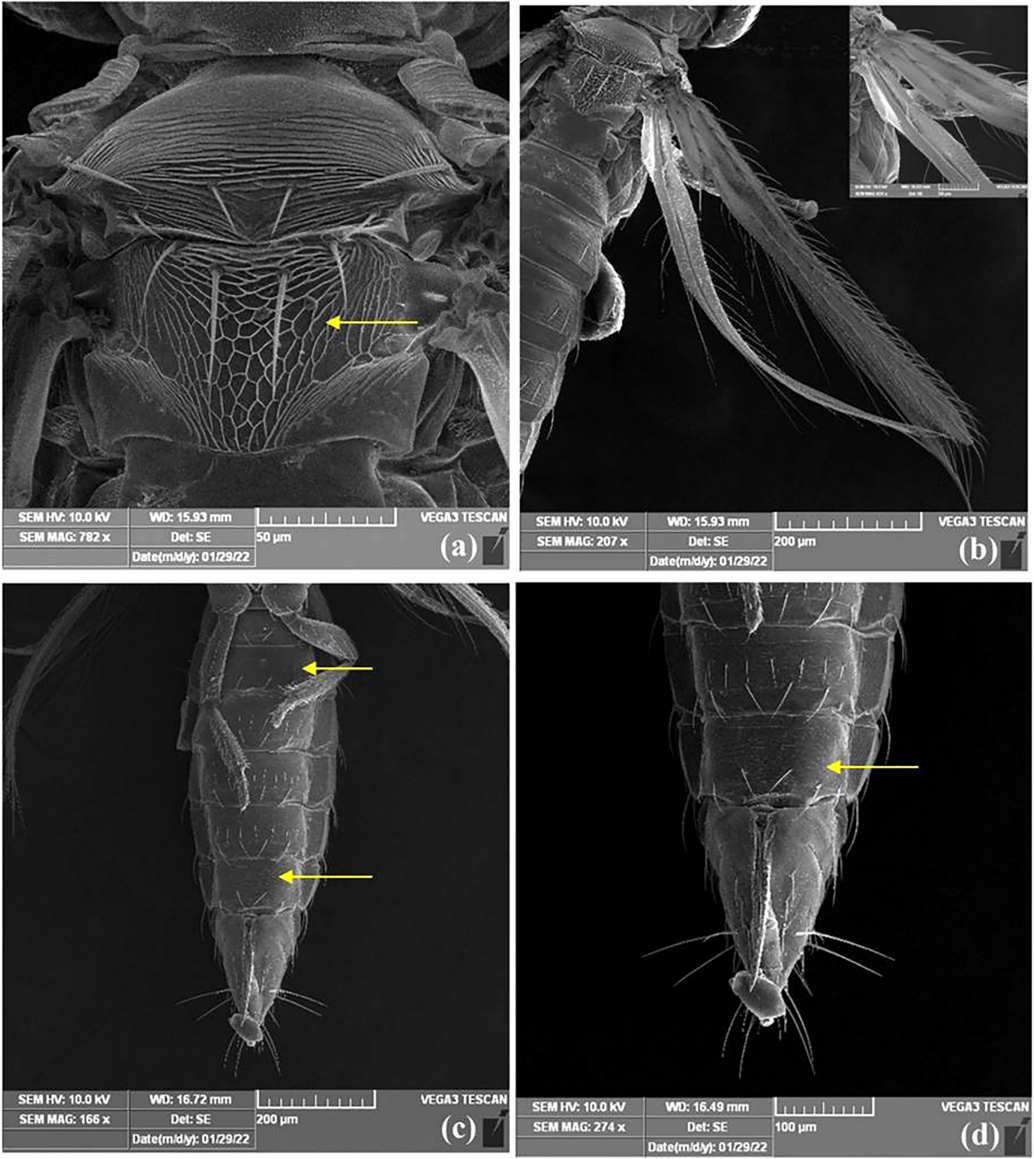
SEM images of *Thrips parvispinus*. (A) Metanotum reticulate medially, median setae long, arising behind anterior margin and campaniform sensilla absent; (B) forewing first and second veins with complete rows of setae and clavus with five marginal setae; (C) sternites II & VII without discal setae, (D) III–VI with about 6–12 discal setae.

Female thrips adults were dark brown, head and thorax paler than abdomen, legs were yellow. Male thrips adults were light brown to yellowish colour and smaller in size as compared to females (Fig. 3). Scanning electron microscopy revealed thrips were having seven segmented antennae (Fig. 4A), forked sensory organs present on III & IV segments (Fig. 4B). Head wider than long, three pairs of ocellar setae, III pair is small and located outside the ocellar triangle (Fig. 4C); postocular setae I and III pair are longer than ocellar setae III. Two pairs of long posteroangular setae and three pairs of posterior marginal setae are present on the pronotum (Fig. 4D). There is no campaniform sensilla on metanotum and reticulates medially; long median setae are located behind the anterior margin (Fig. 5A). First and second veins with two complete rows of setae in the fore wing and five marginal setae located at clavus region (Fig. 5B). Irregular rows of discal setae (6–12) on sternites III–VI, but absent on II & VII (Fig. 5C). Male thrips with yellow colour body and there is no posteromarginal comb on VIII tergite; sternal segments III to VII have small transverse pore plate and discal setae located laterally (Fig. 5D). Material examined. Two females; two males, India: Andhra Pradesh: Rajahmundry, 2022, Devaramane Raghavendra (RRS. No. 277/2022 to 280/2022).

### Molecular diagnosis of *T. parvispinus*

**Figure 6:**
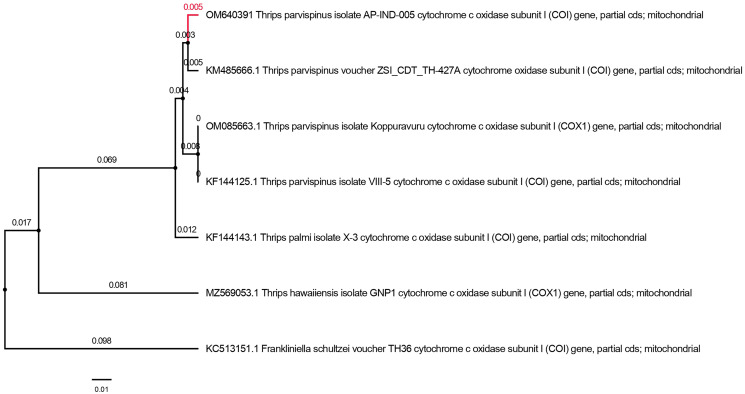
Neighbor-joining tree of *Thrips parvispinus. T. hawaiiensis and F. schultzei* is used as outgroup.

The PCR amplicon of size (700 bp) for mtCOI gene was amplified and the mitochondrial cytochrome oxidase I gene sequencing yielded a nucleotide length of 690 base pair for *Thrips parvispinus*. The sequences obtained were properly trimmed yielding 650–680 base pairs, BLASTn search of *T. parvispinus* in NCBI showed 97.99 to 98.81 per cent similarity with the respective species deposited from Andhra Pradesh (Kopparavuru), Telangana, Karnataka states of India, and Indonesia. The sequences generated in the present study were submitted to NCBI GenBank (accession numbers: OM640388, OM640389, OM640390, OM640391). We analyzed six partial mtCOI sequences of *T. parvispinus* and *T. hawaiiensis* in this study. The analysis of the Neighbor-joining (NJ) tree yielded two major clades with high bootstrap support; clade I included four sequences of *T. parvispinus* from Andhra Pradesh, Telangana, Karnataka (India), and Indonesia, while clade II was represented by one sequence of *T. hawaiiensis* (Fig. 6) and *Frankliniella schultzie*, an outgroup as expected to form a separate clade. According to reciprocal monophyly criteria, NJ tree provided here is only to segregate two species rather than interpreting the phylogeny of genus *Thrips*. The morphological and molecular diagnosis studies confirmed the thrips collected from chilli invaded areas of Andhra Pradesh, Telangana, and Karnataka represent *Thrips parvispinus* Karny. Understanding its invasiveness, the current distribution and future spread of *T. parvispinus* were predicted.

### Important bioclimatic variables for *T. parvispinus* distribution

**Figure 7:**
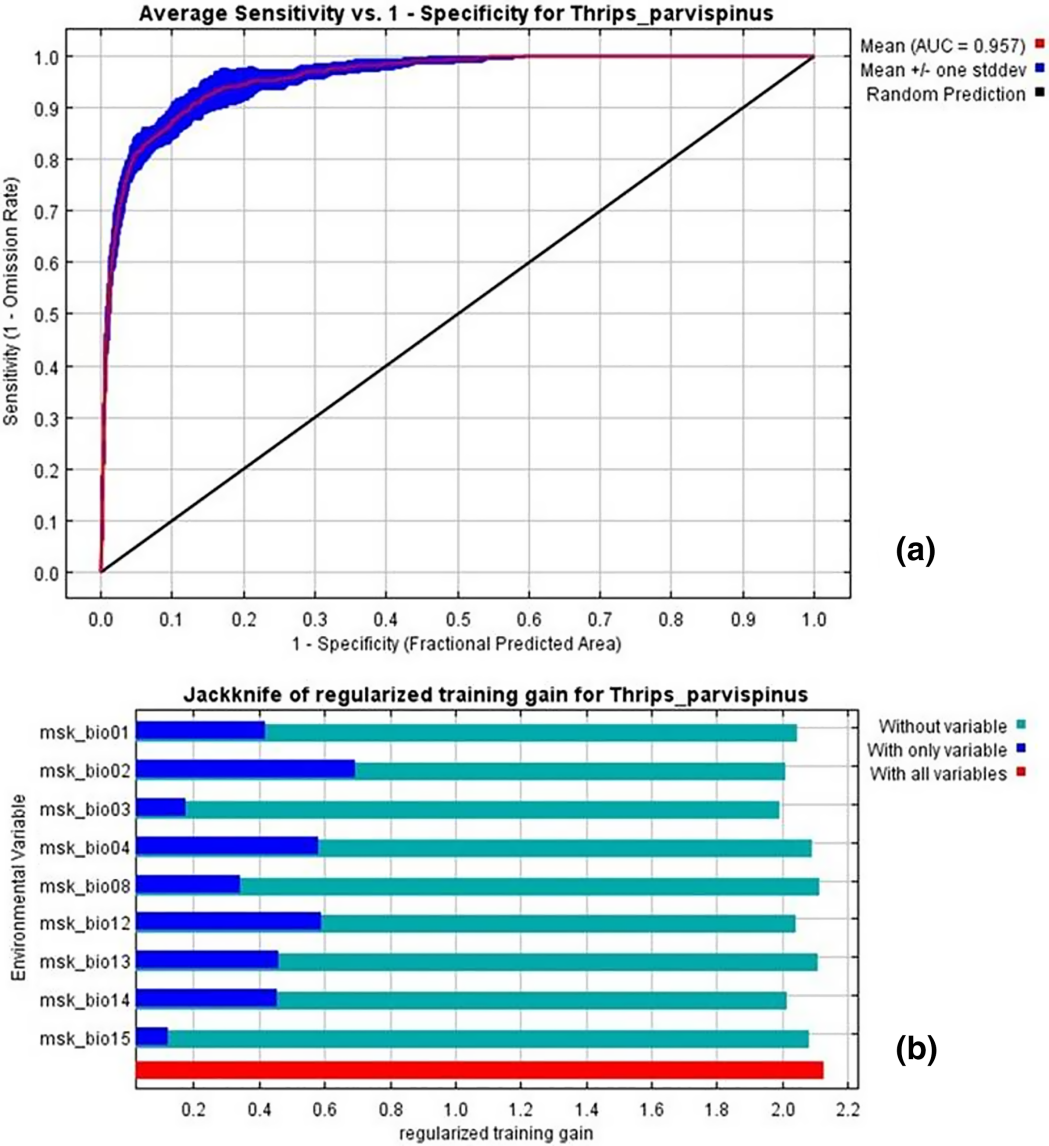
*Thrips parvispinus* under current climatic conditions. (A) Receiver operating characteristic (ROC) curve; (B) jackknife test of variable importance. Graphics show variable contributions to regularized training gain.

**Figure 8:**
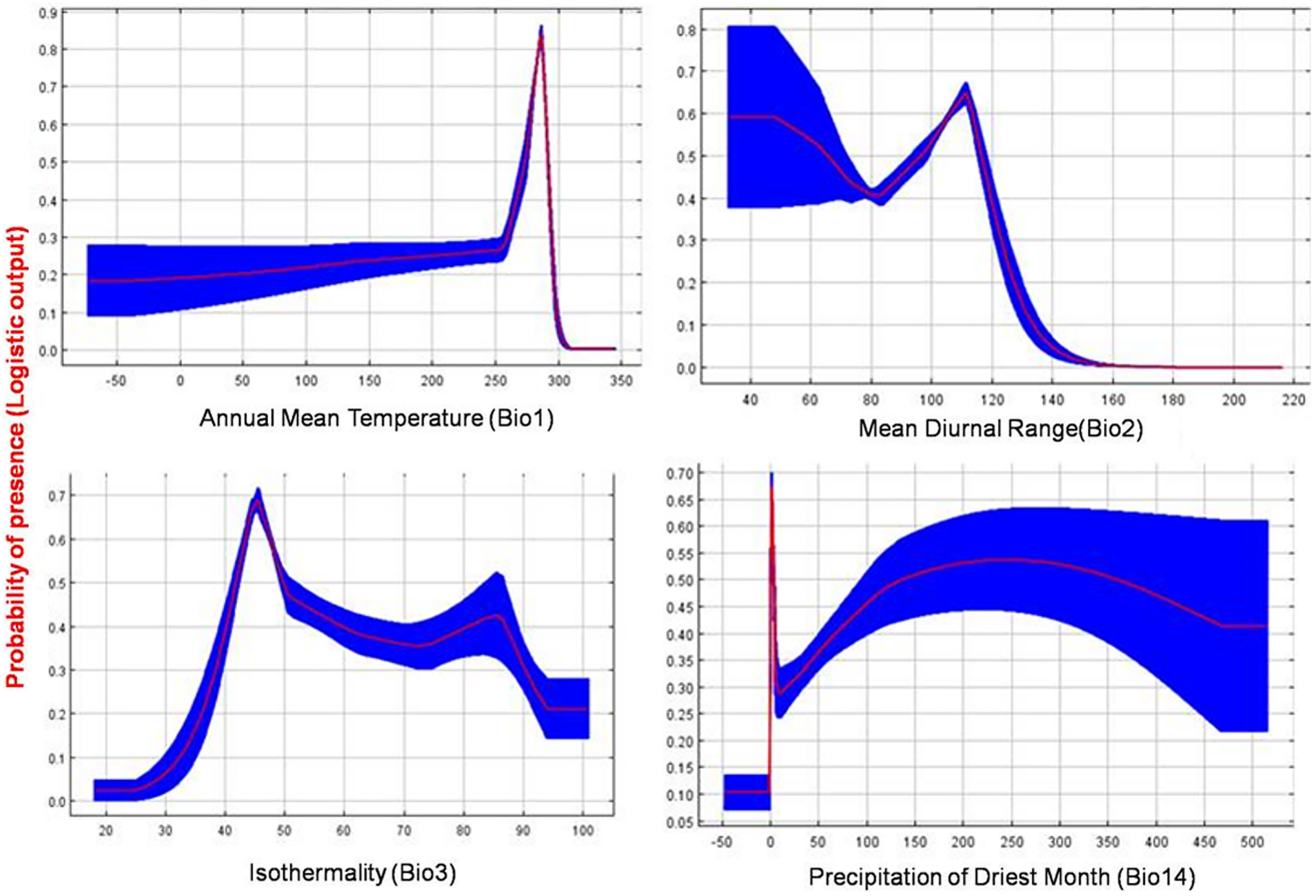
Response curve plots illustrate the dependence of predicted habitat suitability on important environmental variables. The curve shows the mean response of 10 replicates by MaxEnt (red) and the mean ± SD.

The current distribution of *T. parvispinus* satisfactorily with test omission rate close to predicted omission and receiver operating characteristic (ROC) curve, prediction with the average area under curve (AUC) value of 0.957 (Fig. 7A). These results indicated that temperature-related bioclimatic variables had a vital role in determining habitat suitability. The highest gain was observed with the environmental variable, temperature seasonality (Bio2), which therefore appeared to provide the most useful information. Whereas a maximum decrease in the gain on omission was observed with isothermality (Bio3), which appeared to have the most information that was not present in the other variables (Fig. 7B). The relationship between important bioclimatic variables indicated the probability of peak presence of *T. parvispinus* predicted when the annual mean temperature of 25 °C, with the mean diurnal range of 11 °C, isothermality of 4 °C, and precipitation of driest month between 5–50 mm (Fig 8).

### Distribution of *T. parvispinus* under current climate scenarios

**Figure 9:**
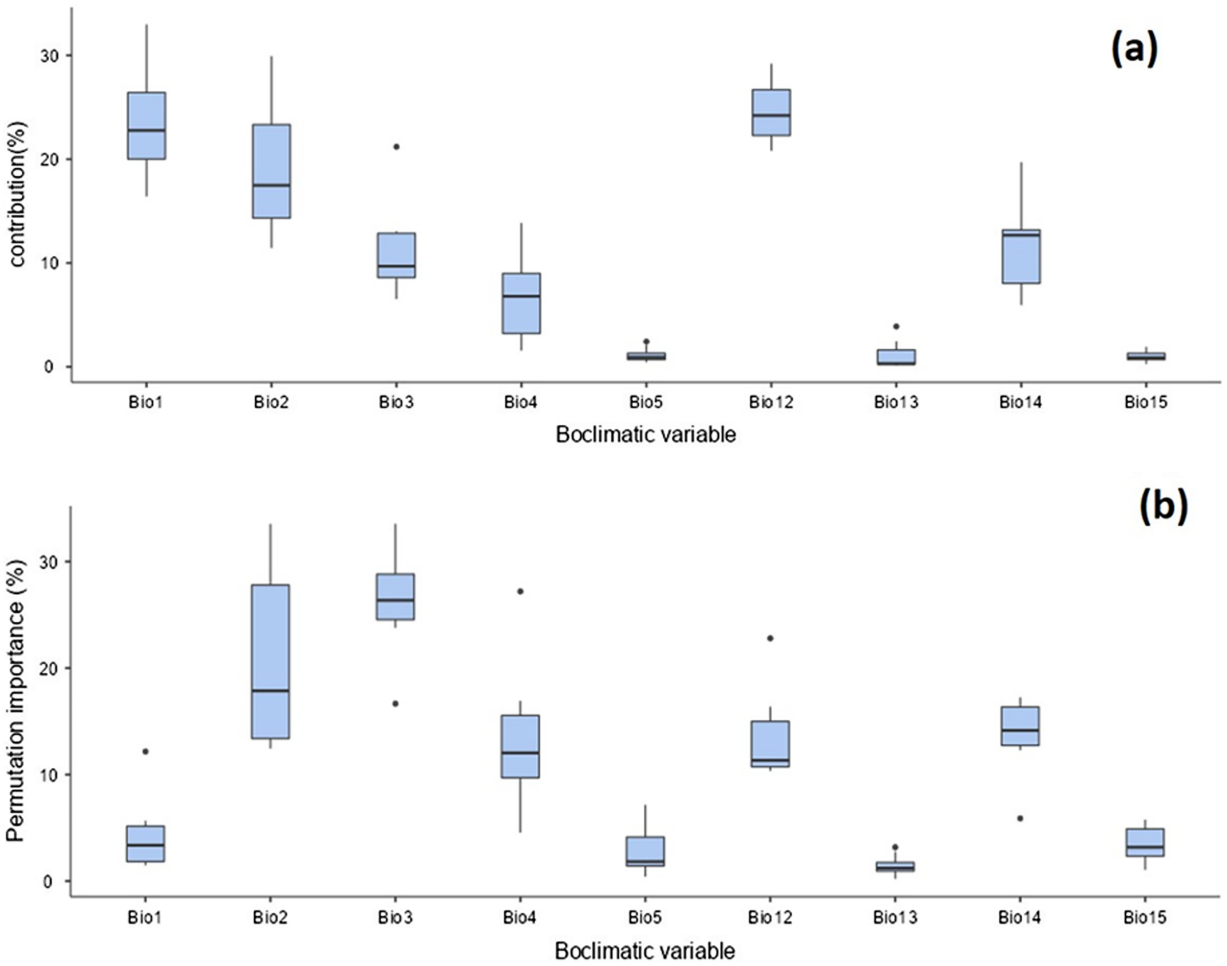
Prediction of *Thrips parvispinus distribution with bioclimatic variables*. (A) Percent contributions; (B) permutation importance.

The distribution of *T. parvispinus* under current climate scenarios was found to be determined by annual precipitation (24.6%), annual mean temperature (23.5%), mean diurnal range (19%), precipitation of the driest month (11.4%), and isothermality (11.1%) (Fig. 9A). Considering the permutation importance of the bioclimatic variables, isothermality (Bio3) was found to be the most important variable in determining the *T. parvispinus* distribution in the model with 27.4% contribution (Fig. 9b).

**Figure 10:**
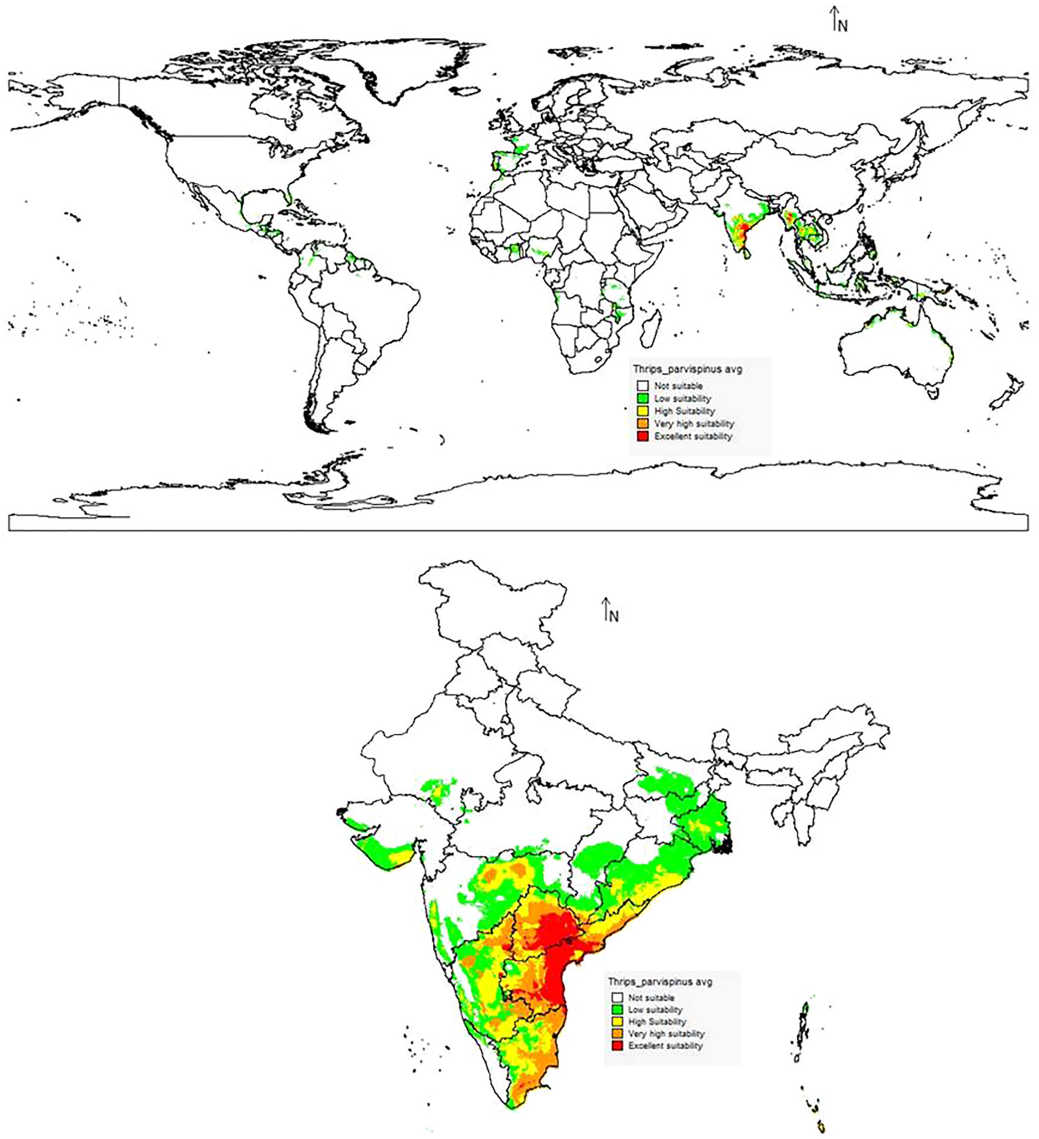
Prediction of distribution of *Thrips parvispinus* under the current scenario.

The suitability discrimination of *T. parvispinus* occurrence indicated was predicted high suitability areas in India, Myanmar, and Thailand. Low to moderate suitability was predicted in other areas (Fig. 10). In India, southern states like Andhra Pradesh, Telangana, the Northern part of Tamil Nadu, and Karnataka were predicted as excellent suitability areas for *T. parvispinus* occurrence (Fig. 10).

### Prediction for *T. parvispinus distribution* under future climate change scenarios

**Figure 11:**
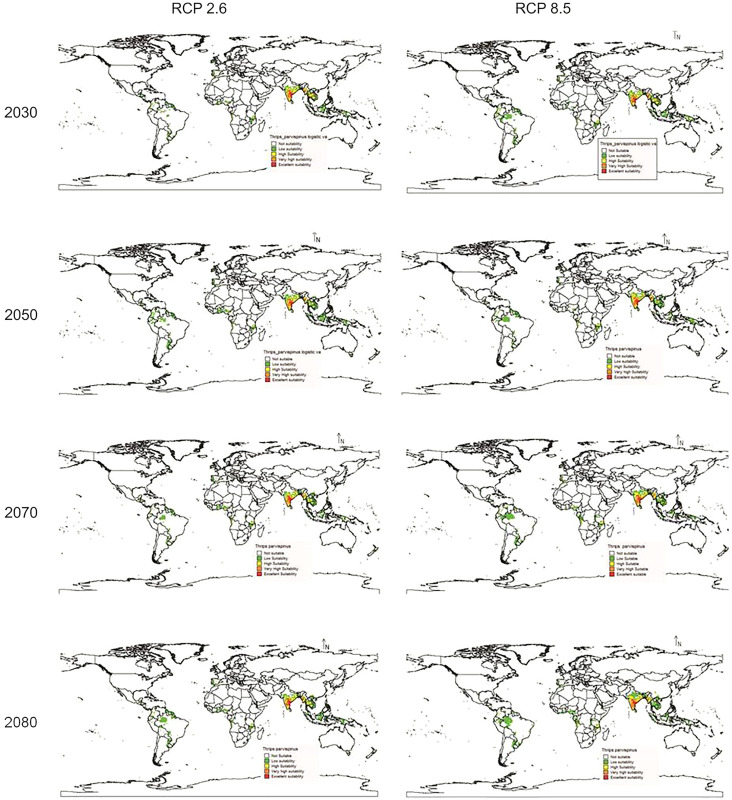
Prediction of distribution of *Thrips parvispinus* under future climate model of Cisro.mk3.

**Figure 12:**
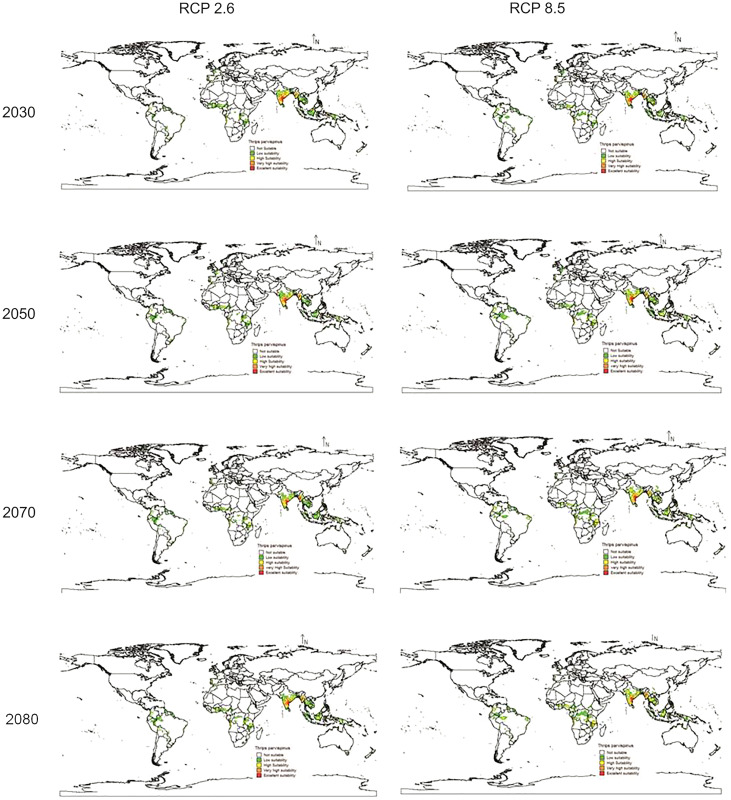
Prediction of distribution of *Thrips parvispinus* under future climate model of GFDL.

**Figure 13:**
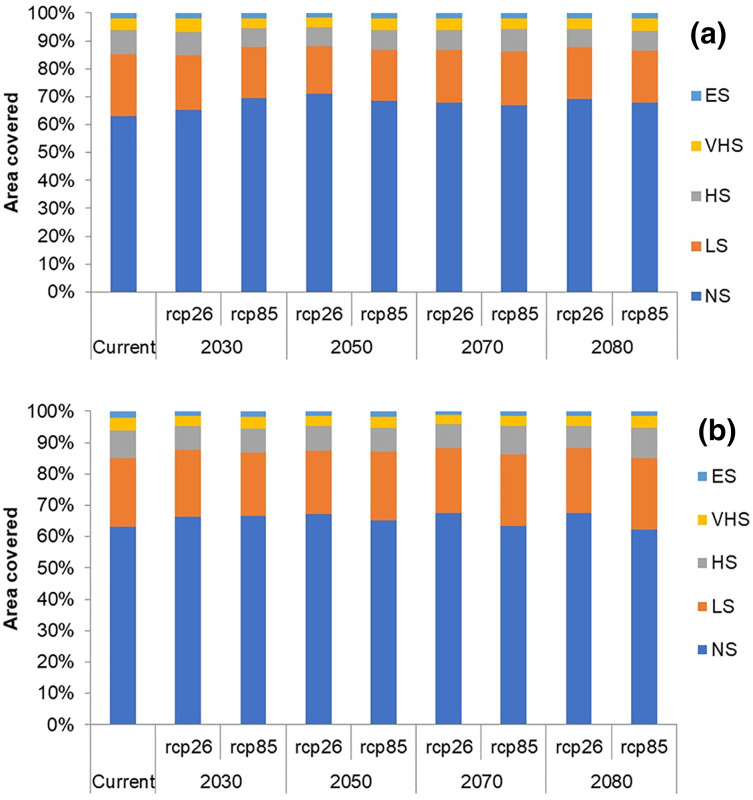
Impact of climate change on the distribution of *T. parvispinus* as predicted in the models. (A) CISRO.MK3 and (B) GFDL (ES, Excellent suitability; VHS, very high suitability; HS, High suitability; LS, less suitability and NS, not suitable).

In future climate scenarios, for the years 2030, 2050, 2070, and 2080, the distribution of *T parvispinus* was predicted to increase relatively in the area from the current distribution. The highest area of excellent suitability was found in the RCP 8.5 (pessimistic estimation) scenario as compared to the RCP 2.6 (optimistic estimation). Excellent suitability areas for pest occurrence in the future climate scenario were predicted in India, Myanamar, Thialand (Figs. 11 and 12). According to CISRO.MK3 model, excellent suitability areas for the occurrence of *T. parvispinus* will be expanded to the tune of 0.1% in RCP 2.6 of 2030, 0.3% in RCP 8.5 scenario of 2050, 0.5% and 0.2% in RCP 2.6 and RCP 8.5 of 2050 and RCP 2.6 of 2080 as compared to the current distribution of *T. parvispinus* (Fig. 13A). Very high suitability areas will be expanded to 0.23% in the scenario of RCP 8.5 of 2080, whereas the GFDL model predicted only an increase in the high suitability areas in the scenarios of RCP 8.5 of 2070 and RCP 8.5 of 2080 up to 0.32% and 0.8% respectively (Fig. 13B). The model predicted there will be up to 0.8% increase in the suitability areas of *T. parvispinus* under future climate.

## Discussion

Invasive pests are one of the major threats in modern agriculture. Efficient management of invasive pests depends on its quick diagnosis, monitoring, and predictionof its current and future invading risks. Failure in any one of these results serious negative impact on crop production and native biodiversity (flora and fauna). One such example is the recent outbreak of invasive *Thrips parvispinus* Karny in south Indian states. This invasive thrips identity was confirmed by both morphological and molecular taxonomy. The key morphological characters of *T. parvispinus* were ascertained with high-resolution scanning electron microscopic images, further MtCOI gene-specific primers were used for the molecular confirmation.

The Maxent model in the current study demonstrated that the temperature-related bioclimatic variables such as annual mean temperature, (BIO 1) diurnal temperature range (BIO 2), and annual precipitation (BIO 12) have higher predictive importance.

In the currently invaded locations of India, temperature and precipitation play a vital role in determining the distribution of *T. parvispinus*. During November and December, the chilli growing areas received low-intensity scattered precipitation (43 to 82 mm) with favourable temperatures (*i.e*., 25 °C to 27 °C), the diurnal temperature was 6 °C to 10 °C. Further, in a more congenial environment recorded during December, the precipitation decreased (0 mm to 42 mm) and the temperature range was 23 °C to 25 °C, the diurnal temperature was in the range of 11 °C to 14 °C (Table S2). This is reflected in the prediction model *i.e*., The high contribution (24.6%), with annual precipitation (BIO 12) followed by temperature (BIO 1) (23.5), and diurnal temperature (BIO 2) (19%). These three bioclimatic variables influenced the movement of thrips from infested to new areas and created a favourable environment for population buildup. To improve our understanding of the invasion risk of insect pests, like *T. parvispinus*, invading tropical and temperate regions, temperature and precipitation might be the most critical factors to determine *T. parvispinus* survival and establishment in introduced new areas.

Since this pest is originated from a similar kind of tropical climate (Indonesia and Thailand), and availability of its host plants (solanaceous and cucurbitaceous crops) may accelerate its adaptability to the Indian agro-climate.

Several studies established the linear relationship between the temperature and generation time of thrips, which may lead to the expansion in the geographical distribution (Tommasini & Maini, 1995; Murai, 2002; Park et al., 2014). Like other invasive pests, *T. parvispinus* has taken a short *lag phase* duration for the establishment and spread to the major chilli growing areas of south India. *Lag* times related to biological invasions of other exotic species exhibited almost a similar trend (Crooks, 2005; Jeffrey, 1999). The maximum reproduction and survival potential of *Thrips parvispinus* was observed at the temperature range of 25 °C to 27 °C in Indonesia (Hutasoit, Triwidodo & Anwar, 2017). Similarly, the temperature ranges from 15 °C to 30 °C, was positively influenced the thrips population in crops like chilli (Meena et al., 2005), groundnut (Vijayalakshmi, 1994), tomato (Hn et al., 2019). The parthenogenetic mode of reproduction of thrips in a short generation time (15 days to 1 month) is another important factor that contributes to the rapid development and spread (Brodsgaard, 1989; Nault et al., 2006).

Validation using independent data observed from India confirmed the suitability of the MaxEnt model at explaining changes to the distribution of *T. parvispinus* (Figs. S1 and S2). Moreover, our results supported a previous study performed with a mechanistic niche model of *T. palmi* (CLIMEX model) (Park et al., 2014).

In the present study, the model predicted increasein the high suitability of current and future climate areas for pest occurrence following the changes in the temperature regimes in the climate change scenarios. Under the current scenario excellent and high suitability areas for the *T. parvispinus* occurrence were predicted in India, Myanmar, Indonesia, and Thailand, Likely distribution pattern of *T. parvispinus* as projected in the future climate scenario varied across the different RCPs from optimistic (RCP 2.6) to pessimistic (RCP 8.5) estimates of climate change.

The probability of the spread of the pest in the conventional areas of its occurrence will be more in the RCP 8.5 scenario.

The high-temperature prediction, which strongly supports the pest’s growth and development, may be to reprimand for this change. As there is inherent uncertainty in modeling anthropogenic climate change, different RCPs are indeed expected to exhibit variability in the adaptation of pests in new locations (Pearson et al., 2006; Thuiller, 2004). Andhra Pradesh, Telangana, and the northern regions of Karnataka and Tamil Nadu in India, as well as Bangladesh, were identified as hotspots for the occurrence of *T. parvispinus*. The results of the present study should be interpreted with care. Maybe there are prediction uncertainties with correlative niche models (Jarnevich et al., 2015). These uncertainties are predominantly due to the consistency of data onpest prevalence, sampling bias, spatial data layer resolution, species characteristics, and spatial autocorrelation (Anderson, 2013). However, the accuracy of the model can be improved significantly with the calibrations of the background points and extent by regularizing parameters with suitable multipliers and selecting suitable feature types (Phillips, Anderson & Schapire, 2006; Kumar et al., 2015). The potential pitfalls in the model were addressed by appropriate calibrations so that predictive models developed in the present study were consistent with the current distribution pattern of the species and it was confirmed with satisfactory validation.

## Conclusions

This is the first study that documented the key distinguishing morphological characters of *T. parvispinus* were illustrated through high-resolution scanning electron microscopic images as well as molecular tools. This makes it easy for researchers to locate and easily differentiate new *Thrips parvispinus* Karny from other thrips species. And also, this is the first study to develop global and local potential invasion risk maps for thrips. MaxEnt model predictions are highly accurate and conformed well to the current known distribution of the pest.

The model predicted species range in respect of discrimination of suitable and unsuitable areas for its occurrence both in current and future climatic scenarios. The model provided a good fit for species distribution with a high value of area under the curve (0.957). The jackknife test indicated annual mean temperature and precipitation were found to be the most important bioclimatic variable in determining the distribution of *T. parvispinus*. High suitability areas were predicted in the countries wherever its occurrence was reported with high discrimination ability of suitable and unsuitable areas. Key distinguishing morphological characters of *T. parvispinus* were illustrated through high-resolution scanning electron microscopic images. MaxEnt model identified high suitability regions for the potential establishment of *T. parvispinus* in India and other parts of the world. This study facilitates forecasting of further spread and also suggests imposing strict domestic quarantine regulations to curtail its spread andestablishment in new areas.

These results can be used by plant protection organizations and pest managers to guide pest risk assessments, and for monitoring inadvertent introductions of *T. parvispinus*. The maps can be used in pest control efforts and in designing future sampling strategies in currently infested areas. These results can also be used by policymakers in developing appropriate phytosanitary measures and management strategies and making science-based international trade decisions.

## Supplemental Information

10.7717/peerj.13868/supp-1 Supplemental Information 1 Supplemental Tables. *Thrips parvispinus* Karny occurrence in different countries with a timeline. Weather data of study locations at South Indian States 2021.

10.7717/peerj.13868/supp-2 Supplemental Information 2 Prediction of distribution of *Thrips parvispinus* in India under future climate model of Cisro.

10.7717/peerj.13868/supp-3 Supplemental Information 3 Prediction of distribution of *Thrips parvispinus* In India under future climate model of GFDL.

10.7717/peerj.13868/supp-4 Supplemental Information 4 Raw data of thrips occurrence at South Indian states.

## Additional Information and Declarations

### DNA Deposition

The following information was supplied regarding the deposition of DNA sequences: Data is available at NCBI GenBank: OM640388, OM640389, OM640390, OM640391.

### Data Availability

The following information was supplied regarding data availability: The raw data is available in the Supplemental Files.
