# Supplementary material for: Diagnosis and potential invasion risk of Thrips parvispinus under current and future climate change scenarios
Source: PeerJ. 2022 Aug 25;10:e13868. doi: 10.7717/peerj.13868 (PMC9420409; doi:10.7717/peerj.13868)
Supplement: Supplemental Information 1 — Thrips parvispinus Karny occurrence in different countries with a timeline. Weather data of study locations at South Indian States 2021. [file peerj-10-13868-s001.docx]

**Supplementary Table 1**. Invasion of Thrips *parvispinus* Karny in different countries with timeline

| Year | Country | Regions | Host plants | Reference |
| --- | --- | --- | --- | --- |
| 1980 | Malaysia | Serdang,  Peninsular Malaysia | *Carica papaya,* capsicum, cucumber, hibiscus rosa, egg plant, vigna , cowpea | Lim, W. H 1989, MARDI, 1980 |
| 1990 | Mauritius Greece | Volos | *Gardenia* | Anagnou-Veroniki et al., (2008) ;  NPPO, 2007 |
| 1991 | Thailand | Kanchanaburi Province |  | Waterhouse, 1993  EPPO, 2021 |
| 1996 | Netherland |  | beans, eggplant, papaya, pepper, potato, shallot and strawberry | NPPO,1996 |
| 1998 | Indonesia | Jawa,Jambi  Borgor, Bali  East Java, Central Java | Chilli, pepper, paprika, green bean, cucumber potato, strawberry, eggplant | Vos & Frinking 1998; ; Murai et al., 2009; Sartiami and Mound 2013;Johari A 2015 |
| 2000 | Africa | Reunioun, Tanzania Uganda, Burundi |  | Bournier J P 2000  Moritz et al., 2013 |
| 2000 | Singapore |  | citrus, melon, pepper, tobacco | EPPO 2000 |
| 2000 | Australia | Broome South Wales Solomon Islands |  | EPPO 2000/061  Mound and Masumoto 2005 |
| 2005 | Taiwan |  |  | Mound and Mosumoto 2005;Majid Mirab-baloa et al., 2011; Zhang et al., 2015 |
| 2006 | Florida | Orange county | *Carica papaya* | Mound et al., 2016 |
|  | USA | Hawaii Puna Hilo Barbados Honolulu | *Carica papaya* | Sugano et al.,2010 |
| 2008 | France | South western parts | *Mandevilla* | Anagnou-veroniki et al., 2008 |
| 2015 | India | Telangana, Andhra Pradesh, Karnataka, Tamil Nadu, Kerala | *Carica papaya.* *Capsicum*, *Tegetus*, Dahlia sp,  *Brugmansia* sp. *Tagetes* sp. *Citrullus lanatus* (Thunb.) *Momordica charantia* L. *Chrysanthemum* sp. *Gossypium* sp. *Mangifera indica* L. *Tamarindus indica* L. | Tyagi et al., 2015  Rachana et al., 2018;  Nagaraju et al.,2021; Rachana et al. 2021 |
| 2015 | Myanmar |  | Mangifera indica | IPCC, 2015 |
| 2019 | Spain |  | Dipladenia, Gardenia and Citrus. | Lacasa et al., 2019  EPPO, 2019 |
| 2020 | Philippines |  | citrus, melon, pepper, tobacco | Reyes, 2020 |

**Supplementary Table 2**. Weather data of study locations at South Indian States (Telangana, Andhra Pradesh and Karnataka) during 2021

| Month | District | RF (Cumulative) | Temperature ◦C | | | | Relative Humidity (%) | | |
| --- | --- | --- | --- | --- | --- | --- | --- | --- | --- |
|  |  |  | Min | Max | Mean | Diurnal Temp range | Morning | Evening | Mean |
| January | Bhadradri | 0 | 15.83 | 17.49 | 16.66 | 1.65 | 39.87 | 94.19 | 67.03 |
|  | Khammam | 0 | 13.13 | 20.25 | 16.69 | 7.11 | 48.63 | 97.70 | 73.16 |
|  | Mahabubabad | 0 | 13.6 | 18.95 | 16.31 | 5.27 | 41.69 | 99.27 | 70.48 |
|  | Warangal | 0 | 13.72 | 19.07 | 16.40 | 5.35 | 43.08 | 99.77 | 71.42 |
|  | Guntur | 0 | 13.15 | 30.51 | 21.83 | 17.35 | 83.70 | 53.70 | 68.70 |
|  | Raichur | 1.2 | 18.47 | 30.72 | 24.59 | 12.25 | 84.21 | 39.07 | 61.64 |
| February | Bhadradri | 0 | 17.40 | 17.25 | 17.32 | -0.15 | 30.37 | 90.77 | 60.57 |
|  | Khammam | 0 | 14.98 | 19.15 | 17.07 | 4.175 | 40.73 | 96.88 | 68.81 |
|  | Mahabubabad | 0 | 14.99 | 18.54 | 16.76 | 3.54 | 33.59 | 98.25 | 65.92 |
|  | Warangal | 1.2 | 15.20 | 18.01 | 16.60 | 2.81 | 34.37 | 98.68 | 66.52 |
|  | Guntur | 8 | 18.85 | 32.19 | 25.52 | 13.33 | 90.89 | 59.46 | 75.17 |
|  | Raichur | 0 | 18.42 | 32.32 | 25.37 | 13.89 | 69.91 | 25.65 | 47.78 |
| March | Bhadradri | 0 | 16.86 | 21.19 | 19.02 | 4.33 | 27.98 | 88.31 | 58.15 |
|  | Khammam | 0 | 14.95 | 21.30 | 18.12 | 6.34 | 39.21 | 96.98 | 68.10 |
|  | Mahabubabad | 0 | 15.12 | 21.03 | 18.08 | 5.91 | 30.29 | 95.14 | 62.71 |
|  | Warangal | 0 | 15.16 | 21.14 | 18.15 | 5.97 | 30.76 | 95.69 | 63.23 |
|  | Guntur | 0 | 19.86 | 37.6 | 28.73 | 17.73 | 88.37 | 49.35 | 68.86 |
|  | Raichur | 0.6 | 21.83 | 36.87 | 29.35 | 15.04 | 54.05 | 17.41 | 35.73 |
| April | Bhadradri | 0 | 25.89 | 38.58 | 32.24 | 12.68 | 25.09 | 56.50 | 40.80 |
|  | Khammam | 0 | 25.01 | 39.16 | 32.09 | 14.14 | 38.27 | 91.38 | 64.82 |
|  | Mahabubabad | 0 | 23.88 | 37.82 | 30.85 | 13.93 | 32.29 | 85.19 | 58.74 |
|  | Warangal | 0 | 23.68 | 37.70 | 30.69 | 14.02 | 34.45 | 93.60 | 64.02 |
|  | Guntur | 41.30 | 20.48 | 37.82 | 29.15 | 17.33 | 83.17 | 61.12 | 72.14 |
|  | Raichur | 0.8 | 25.25 | 38.49 | 31.87 | 13.24 | 56.51 | 23.71 | 40.11 |
| May | Bhadradri | 146.6 | 25.89 | 35.59 | 30.74 | 9.70 | 51.56 | 89.66 | 70.61 |
|  | Khammam | 504 | 26.59 | 39.05 | 32.82 | 12.46 | 26.46 | 57.87 | 42.17 |
|  | Mahabubabad | 179.6 | 26.32 | 38.62 | 32.47 | 12.29 |  |  |  |
|  | Warangal | 35.9 | 26.44 | 39.00 | 32.72 | 12.56 | 25.37 | 57.70 | 41.53 |
|  | Guntur | 82 | 20.2 | 37.04 | 28.62 | 16.84 | 74.96 | 51.27 | 63.121 |
|  | Raichur | 61.8 | 25.44 | 37.46 | 31.45 | 12.021 | 74.57 | 37.25 | 55.91 |
| June | Bhadradri | 247 | 25.3 | 31.88 | 28.59 | 6.58 | 70.4 | 96.79 | 83.59 |
|  | Khammam | 707.6 | 26.40 | 35.66 | 31.03 | 9.25 | 54.96 | 92.88 | 73.92 |
|  | Mahabubabad | 858.5 | 25.40 | 34.25 | 29.83 | 8.85 | 58.47 | 94.46 | 76.46 |
|  | Warangal | 851.3 | 24.99 | 34.27 | 29.63 | 9.28 | 48.25 | 98.62 | 73.43 |
|  | Guntur | 134.2 | 20.79 | 35.2 | 27.99 | 14.40 | 83.1 | 51.3 | 67.2 |
|  | Raichur | 109.6 | 23.87 | 33.89 | 28.88 | 10.01 | 76.14 | 47.14 | 61.64 |
| July | Bhadradri | 343.8 | 25.05 | 32.92 | 28.99 | 7.87 | 68.38 | 95.74 | 82.06 |
|  | Khammam | 1105.1 | 25.14 | 33.27 | 29.21 | 8.13 | 70.25 | 97.24 | 83.75 |
|  | Mahabubabad | 1698.4 | 24.22 | 31.78 | 28.00 | 7.55 | 70.46 | 97.31 | 83.88 |
|  | Warangal | 1390.6 | 24.79 | 32.05 | 28.4 | 7.26 | 72.60 | 98.84 | 85.72 |
|  | Guntur | 169.2 | 20.17 | 31.53 | 25.85 | 11.3 | 91.58 | 66.41 | 79 |
|  | Raichur | 132.6 | 23.14 | 31.62 | 27.38 | 8.47 | 87 | 60.78 | 73.89 |
| August | Bhadradri | 646 | 24.92 | 31.70 | 28.31 | 6.78 | 78.36 | 99.79 | 89.08 |
|  | Khammam | 586.3 | 25.47 | 33.82 | 29.65 | 8.34 | 67.45 | 96.94 | 82.2 |
|  | Mahabubabad | 540 | 25.23 | 32.59 | 28.91 | 7.36 | 70.43 | 96.54 | 83.48 |
|  | Warangal | 485 | 25.22 | 33.28 | 29.25 | 8.06 | 67.55 | 97.70 | 82.63 |
|  | Guntur | 264.2 | 23.69 | 31.8 | 27.74 | 8.10 | 95.51 | 70.54 | 83.03 |
|  | Raichur | 96.6 | 23.31 | 32.09 | 27.70 | 8.77 | 84.22 | 54.97 | 69.6 |
| September | Bhadradri | 287.6 | 23.90 | 33.63 | 28.77 | 9.72 | 60.34 | 98.95 | 79.64 |
|  | Khammam | 908.7 | 25.02 | 32.98 | 29.00 | 7.96 | 71.19 | 98.74 | 84.96 |
|  | Mahabubabad | 786.2 | 24.59 | 32.29 | 28.44 | 7.69 | 71.54 | 98.45 | 85.00 |
|  | Warangal | 1061.8 | 24.28 | 31.92 | 28.10 | 7.64 | 74.88 | 99.67 | 87.28 |
|  | Guntur | 154.6 | 24.56 | 30.49 | 27.52 | 5.92 | 96.26 | 73.46 | 84.86 |
|  | Raichur | 86 | 22.47 | 30.42 | 26.45 | 7.95 | 89.07 | 64.39 | 76.73 |
| October | Bhadradri | 188.9 | 23.90 | 33.63 | 28.77 | 9.72 | 60.34 | 98.95 | 79.64 |
|  | Khammam | 195.7 | 24.18 | 34.42 | 29.30 | 10.23 | 56.91 | 98.47 | 77.69 |
|  | Mahabubabad | 87.7 | 22.99 | 33.20 | 28.1 | 10.20 | 58.52 | 95.95 | 77.24 |
|  | Warangal | 105.2 | 23.4 | 33.08 | 28.24 | 9.68 | 64.47 | 97.42 | 80.95 |
|  | Guntur | 146.2 | 24.25 | 31.03 | 27.64 | 6.78 | 94.32 | 69.74 | 82.03 |
|  | Raichur | 109.8 | 21.16 | 32.29 | 26.72 | 11.12 | 85.28 | 51.67 | 68.48 |
| November | Bhadradri | 81.7 | 22.45 | 31.39 | 26.92 | 8.94 | 64.78 | 98.38 | 81.58 |
|  | Khammam | 75.5 | 22.50 | 31.75 | 27.12 | 9.24 | 64.01 | 98.42 | 81.22 |
|  | Mahabubabad | 43.2 | 21.26 | 31.70 | 26.48 | 10.43 | 60.96 | 97.96 | 79.46 |
|  | Warangal | 71.1 | 22.40 | 31.64 | 27.02 | 9.24 | 64.42 | 98.13 | 81.28 |
|  | Guntur | 80.50 | 21.95 | 28.20 | 25.08 | 6.25 | 92.90 | 83.10 | 88.00 |
|  | Raichur | 42 | 20.15 | 30.25 | 25.20 | 10.09 | 86.71 | 56.08 | 71.4 |
| December | Bhadradri | 8.2 | 17.38 | 30.78 | 24.08 | 13.39 | 46.24 | 96.84 | 71.54 |
|  | Khammam | 1 | 18.14 | 32.30 | 25.22 | 14.16 | 46.46 | 98.33 | 72.40 |
|  | Mahabubabad | 0.4 | 17.13 | 30.76 | 23.95 | 13.63 | 47.93 | 98.18 | 73.06 |
|  | Warangal | 0 | 17.13 | 30.48 | 23.81 | 13.34 | 40.64 | 97.52 | 69.08 |
|  | Guntur | 3.8 | 17.82 | 28.60 | 23.21 | 10.78 | 97.67 | 61.64 | 79.66 |
|  | Raichur | 42 | 17.04 | 30.70 | 23.87 | 13.65 | 82.92 | 41.86 | 62.39 |
