# Supplementary figures and images for: Diagnosis and potential invasion risk of Thrips parvispinus under current and future climate change scenarios
Source: PeerJ. 2022 Aug 25;10:e13868. doi: 10.7717/peerj.13868 (PMC9420409; doi:10.7717/peerj.13868)

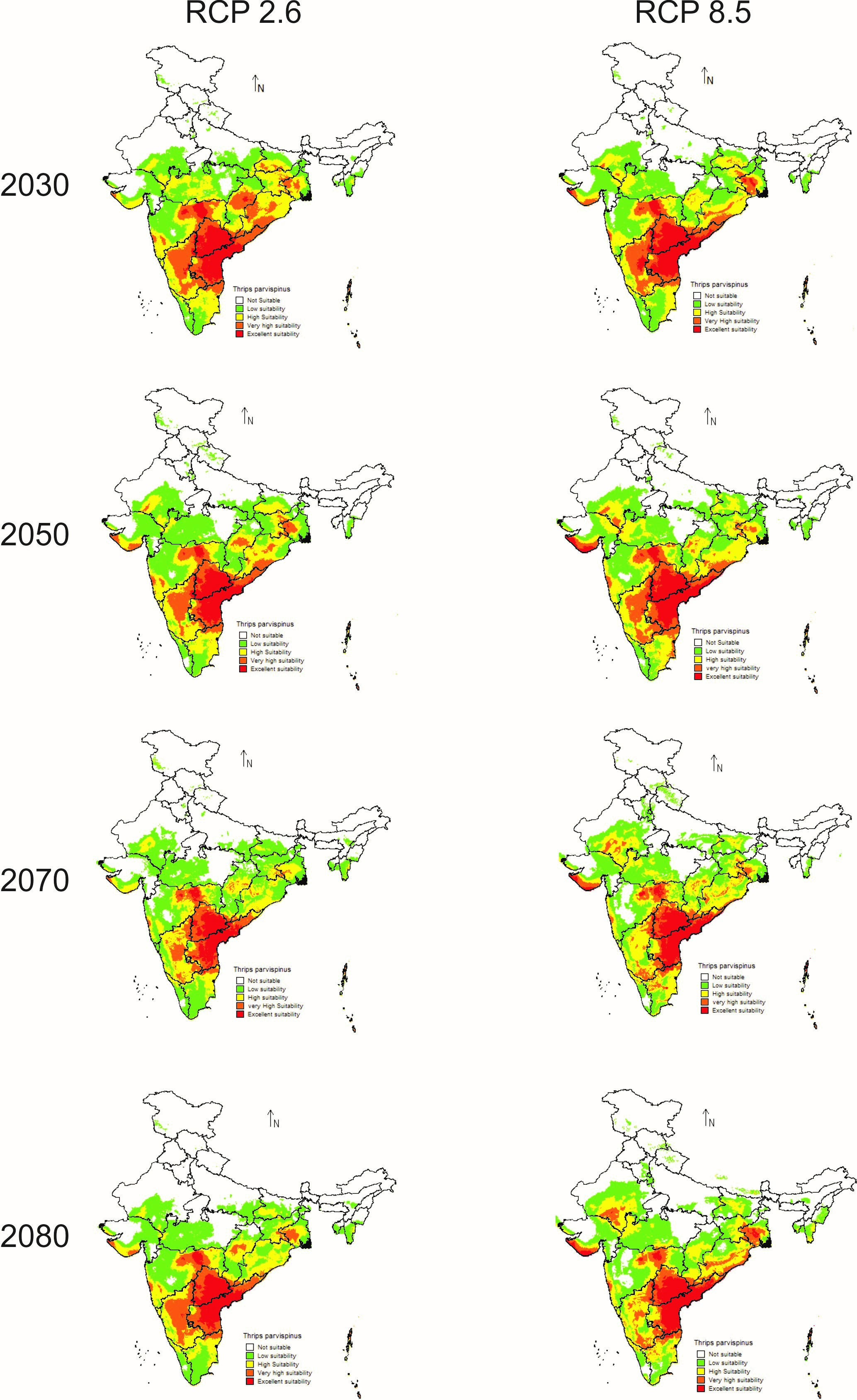

Supplement: Supplemental Information 2 [file peerj-10-13868-s002.jpg]

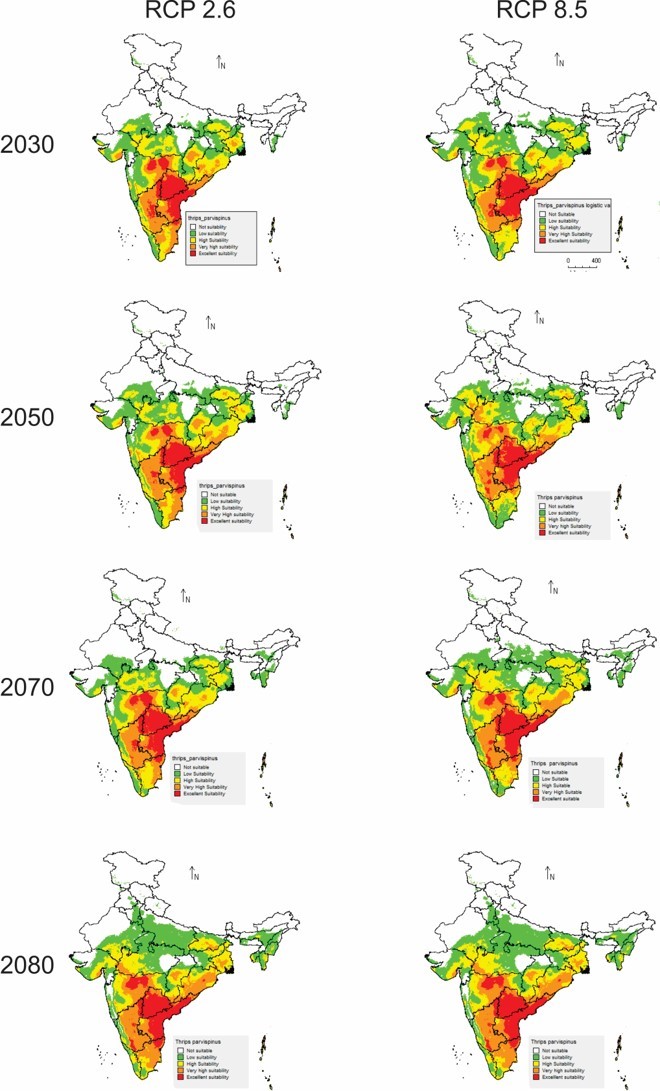

Supplement: Supplemental Information 3 [file peerj-10-13868-s003.jpg]
